# The Disorders of Menstruation

**Published:** 1886-09

**Authors:** 


### Reviews.

**The Disorders of Menstruation.** A practical treatise, by JOHN N. UPSHUR, M. D., Professor of Materia Medica and Therapeutics in the Medical College of Virginia. New York and London: G. P. Putnam's Sons. 1886.

This is a little book of 200 pages, but it contains a great deal of instruction, and is written in a very attractive style. It is intended as a " Student's Manual," but there are few practicing Physicians who will not read it with interest and profit.

